# Coronavirus Genomes and Unique Mutations in Structural and Non-Structural Proteins in Pakistani SARS-CoV-2 Delta Variants during the Fourth Wave of the Pandemic

**DOI:** 10.3390/genes13030552

**Published:** 2022-03-21

**Authors:** Muhammad Zeeshan Anwar, Madeeha Shahzad Lodhi, Muhammad Tahir Khan, Malik Ihsanullah Khan, Sumaira Sharif

**Affiliations:** Institute of Molecular Biology and Biotechnology (IMBB), The University of Lahore, 1 KM Defence Road, Lahore 58 810, Pakistan; dr.zshan@ckmc.edu.pk (M.Z.A.); muhammad.tahir8@imbb.uol.edu.pk (M.T.K.); ihsan.ullah@imbb.uol.edu.pk (M.I.K.); sumaira.sharif@imbb.uol.edu.pk (S.S.)

**Keywords:** SARS-CoV-2, genome, mutations, Pakistan, variants, NSP

## Abstract

Genomic epidemiology of SARS-CoV-2 is imperative to explore the transmission, evolution, and also pathogenicity of viruses. The emergence of SARS-CoV-2 variants of concern posed a severe threat to the global public health efforts. To assess the potential consequence of these emerging variants on public health, continuous molecular epidemiology is of vital importance. The current study has been designed to investigate the major SARS-CoV-2 variants and emerging mutations in virus structural and non-structural proteins (NSP) during the fourth wave in September 2021 from the Punjab province of Pakistan. Twenty SARS-CoV-2 positive samples have been collected from major cities were subjected to next-generation sequencing. Among the 20 whole genomes (GenBank Accession SRR16294858-SRR16294877), 2 samples failed to be completely sequenced. These genome sequences harbored 207 non-synonymous mutations, among which 19 were unique to GISAID. The genome sequences were detected: Delta 21I, 21J variants (B.1.617.2). Mutation’s spike_F157del, spike_P681R, spike_T478K, spike_T19R, spike_L452R, spike_D614G, spike_G142D, spike_E156G, and spike_R158del have been detected in all samples where K1086Q, E554K, and C1250W were unique in spike protein. These genomic sequences also harbored 129 non-synonymous mutations in NSP. The most common were NSP3_P1469S (N = 17), NSP3_A488S (N = 17), NSP3_P1228L (N = 17), NSP4_V167L (N = 17), NSP4_T492I (N = 17), NSP6_T77A (N = 17), NSP14_A394V (N = 17), NSP12_G671S (N = 18), and NSP13_P77L (N = 18). The mutation, F313Y in NSP12, detected in the current study, was found in a single isolate from Belgium. Numerous other unique mutations have been detected in the virus papain-like protease (NSP3), main protease (NSP5), and RNA-dependent RNA polymerase (NSP12). The most common non-synonymous mutations in the spike protein were subjected to stability analysis, exhibiting a stabilizing effect on structures. The presence of Delta variants may affect therapeutic efforts and vaccine efficacy. Continuous genomic epidemiology of SARS-CoV-2 in Pakistan may be useful for better management of SARS-CoV-2 infections.

## 1. Background

The deadly SARS-CoV-2 (Severe Acute Respiratory Syndrome Coronavirus-2) posed a major effect on public health, disrupting the global healthcare system. SARS-CoV-2 has the ability to exhibit a high mutation frequency due to the presence of single-stranded RNA, posing major issues to public health. Molecular epidemiology is required to study the virus evolutionary stages in specific geographic locations and also to establish how the virus may affect the vaccine efficacy and drug response. 

The virus genome encodes 4 structural and 16 non-structural proteins (NSP) [1]. Variations have been reported, occurring very quickly, displacing the virus ancestral strains [2,3,4,5]. At population level, the virus rapid rates of transmission offered an advantage to its replication. The D614G mutation in spike (S) was one of the first discovered, improves viral infectivity, and shifts the spike (S) protein conformation toward binding a fusion-competent state [2,6,7]. Inside the virus S protein, all the variants of concern (VOC) harbored numerous mutations, in the receptor-binding domain (RBD) and in the N-terminus. These VOCs include α (B.1.1.7) (first reported in UK), β (B.1.351) (South Africa), and γ (P.1) in Brazil [4,5,6,7,8]. The Delta variant (B.1.617.2) in India, harbored numerous mutations in RBD SARS-CoV-2 isolates in October 2020 [9]. The latest variant, Omicron (B.1.1.529), was first reported from South Africa on 24 November 2021.

Among the several highly contagious VOCs, a large number of mutations have been detected. The B.1.1.7 lineage has been thought to be 40–80% more contagious [10,11]. However, as compared to P.1 and B.1.351, Delta is exhibiting more transmissible potency than the earlier ones. The VOCs have greater potential in terms of pathogenicity, virulence, and transmission rate, and also exhibit lower antibody neutralization sensitivity [10,12]. Multiple mutations in the S protein and other critical genomic regions of SARS-CoV-2 are resulting in reduced vaccinal response and treatment efficacy. The E484K mutation, present in the receptor-binding ridge, has been detected in S protein in multiple lineages. This mutation has been shown to be decreasing the virus binding to polyclonal sera [13] and protects the virus against treatment with the monoclonal antibodies [14] (p. 19). The P681H mutations have been found in the B.1.1.7, B.1.1.318, and P.3, whereas mutation P681R has been found in the A.23.1 lineages and all B.1.617 variants. The P681H and P681R improve S protein fusion to the host cell [15,16]. The P681H and D614G mutations have been thought to be responsible for the B.1.1.7 increased transmissibility [17]. 

Among the different SARS-CoV-2 variants, B.1.617 lineage was discovered in India [18,19,20]. This lineage includes three primary subtypes (B1.617.1, B.1.617.2, and B.1.617.3), each of which has mutations in the S protein’s N-terminal domain (NTD) and receptor-binding domain (RBD), possesses the ability to boost the immune evasion capability. The Delta variant is thought to spread more quickly than other variants. The B.1.617.1 is characterized by mutations L452R, P681R, and E484Q in S, whereas the Delta variant is characterized by the presence of mutations L452R, P681R, and T478K in S. The L452R and T478K enhance S protein’s binding affinity with human angiotensin-converting enzyme 2 (ACE2) receptor [21,22].

To ensure a better public health policy, it is critical to continuously monitor and identify the rapidly evolving variations in circulating isolates of SARS-CoV-2 in populations. The current study was designed to investigate the major circulating VOC and also the emerging mutations in structural proteins and NSP among the SARS-COV-2 patients from major cities in the Punjab province of Pakistan. Twenty SARS-CoV-2 positive samples have been collected and whole-genome sequencing was performed through Ion Torrent next-generation technology. All the samples harbored numerous mutations in NSP and structural proteins, including S protein. 

## 2. Materials and Methods

### 2.1. Ethical Approval

The ethical clearance for the current study was obtained from the Ethical Review Committee for Medical and Biomedical Research at The University of Lahore (IMBB/UOL/21/1379).

### 2.2. Area of Sample Collection

The nasopharyngeal swab method was used to collect samples from COVID-19 infected individuals, as recommended by the World Health Organization (WHO) [23]. All the samples were collected for whole genome sequencing during the fourth wave in September 2021 from major cities in Punjab province, including Lahore, Sialkot, Okara, and Pak Pattan. 

### 2.3. Processing of Samples

The proper specimen collection procedure under laboratory testing for coronavirus disease (COVID-19) interim guidance by WHO was followed for SARS-CoV-2 samples collection and processing. The nasopharyngeal swabs were collected in 2 mL of vial transport medium (LinkGen, Taizhou, Jiangsu, China) and placed at 4 °C for further use. All these samples were confirmed with TaqPath™ COVID-19 CE-IVD RT-PCR Kit (Thermo Fisher, Waltham, MA, USA).

### 2.4. RNA Extraction, Quantification and cDNA SYNTHESIS

RNA extraction was performed from nasopharyngeal swabs using a standard volume of 200 ul by MagMAX™ Viral/Pathogen Nucleic Acid Isolation Kit (Thermo Fisher, Waltham, MA, USA) and (Applied Biosystem, Waltham, MA, USA). The extracted RNA was stored at −80 °C for downstream application. The real-time quantification and copy number determination of nucleic acid was performed using TaqPath™ 1-Step RT-qPCR Master Mix (Thermo Fisher, Waltham, MA, USA) and the relevant copy number was determined according to the CT and control reactions in quantification. Only samples with relevant CT of 18–28 values were chosen. Using a minimum input amount of 10 ng/uL of the RNA sample, RNA to cDNA reversed transcription was performed with the SuperScript™ VILO™ cDNA Synthesis Kit (Thermo Fisher, Waltham, MA, USA).

### 2.5. Library Preparation

The library was prepared manually using Ion AmpliSeq™ Library Kit (Thermo Fisher, Waltham, MA, USA). Amplification was performed with a 2-pool Ion AmpliSeq™ SARS-CoV-2 Research Panel (Thermo Fisher, Waltham, MA, USA). The library was partially digested and ligated with Ion Xpress™ Barcode Adapters 1–96 Kit (Thermo Fisher, Waltham, MA, USA). Library purification and quantification steps were carried out with GenDx-AMPure® XP beads (GENDX, Utrecht, The Netherlands) and TaqMan™ Quantitation Kit (Thermo Fisher, Waltham, MA, USA). The libraries were diluted to a final concentration of 40 pM, loaded on Thermo Fisher Scientific Ion Chef™ Instrument for PCR, and loaded on Ion S5 530 and 510 (Thermo Fisher, Waltham, MA, USA). 

### 2.6. Whole Genome Sequencing and Data Analysis

Sequencing was accomplished on Ion GeneStudio™ S5 System at LabGenetix, Lahore, Pakistan. The fastQ base sequence file quality was assessed using the FastQC (v0.11.8). Trimmomatic tool (v0.39) was used to remove the low-quality reads (Q < 30) and index adapter sequences were utilized to improve sample multiplexing. Sequenced reads were aligned with the reference (NC 045512, using Burrows Wheeler Aligner (BWA, v0.6). The duplicated PCR reads were removed with Picard Tools (v2.21.6). Mapping problems due to the presence of small Indels were removed with a Genome Analysis Toolkit, “RealignerTargetCreator” and “InDelRealigner” (GATK v. 3.3.0) to analyze the mapped read. To improve the accuracy of variant calling, GATK tool “HaplotypeCaller” was applied for realignment of sequenced reads via local de-novo assembly of haplotypes in the regions showing variation. 

All the 20 whole genome sequences in fasta format were aligned with reference (NC_045512) using CoVsurver application (https://www.gisaid.org/epiflu-applications/covsurver-mutations-app/ (accessed on 28 September 2021)) on 15–20 September 2021, collected during the fourth wave of infection. The CoVsurver research tool has been developed with GISAID (Global initiative on sharing all influenza data), aiding researchers to identify and interpret the amino acids (aa) changes in coronavirus genomes. The server rapidly aligns the query genome sequences in fasta format with reference SARS-CoV-2 and screen coronavirus genomes for aa changes to identify any special epidemiological relevance. All mutations in structural proteins of SARS-CoV-2 were separated and arranged in the form of excel sheets. The statistical analysis was performed using EpiData Analysis [24] to analyze various non-synonymous mutations in virus structural and NSP. 

### 2.7. Mutations Effect on Virus Structural Proteins

Some of the most common mutations were analyzed for their thermodynamic effect on S protein through DynaMut server [25]. The server implements mutation effect, which can be used to analyze the protein stability and structural flexibility upon point mutation. The server also measures the vibrational entropy changes and the impact of a mutation with graph-based signatures (*p*-value < 0.001) along with a good resolution of results. To compute the SARS-CoV-2 wild type and mutant protein stability and flexibility, the PDB file of proteins were retrieved from the Protein Data Bank [26] and uploaded to the DynaMut server and a point mutation was inserted at specific site. The impact in the form of total energies and vibrational entropy energies between wild type and mutants was recorded. The high-resolution structures of wilt type and mutant S proteins were retrieved for further processing.

In DynaMut, changes upon point mutation on free energy of protein folding, combining the impacts of mutation stability of protein and also the dynamic properties were computed by ENCoM, Bio3D, and DUET computational servers, generating a more robust predictor of energies.

### 2.8. Phylogenetic Analysis

Genomic sequences in the current study were subjected to MAFT server [27] and Nextstrain [28] for phylogenetic analysis. These are public servers, containing a pipeline for analysis, and visualization, presenting a real-time view into the evolution of viral pathogens. 

## 3. Results

### 3.1. SARS-CoV-2 Patient Information

Twenty nasopharyngeal swab samples were collected during the fourth wave of the pandemic in September 2021 from SARS-CoV-2 patients (Table 1). Among these samples, 13 were collected from male suspect and seven from female. All the SARS-CoV-2 patients developed clear signs and symptoms, including fever, cough, headache, fatigue, and also significant loss of smell and taste. Eleven patients were found in age category 1 (20–40), seven were in category 2 (41–60) and two were 65 years old.

### 3.2. Whole Genome Sequences

Among the 20 samples (GenBank Accession No. SRR16294858-SRR16294877), samples 7 and 10 (Table 2) (Accession No. SRR16294864 and SRR162948667) were not completely sequenced. The genomic sequences were submitted to NCBI BioProject PRJNA770504 (https://www.ncbi.nlm.nih.gov/bioproject/PRJNA770504 (accessed on 28 September 2021)). The average length of sequenced genomes was 29,586 nucleotides (9691 amino acids). The sequence information, including length and clade, is shown in Table 2. 

### 3.3. Unique Mutations in Structural Proteins

**Figure 1:**
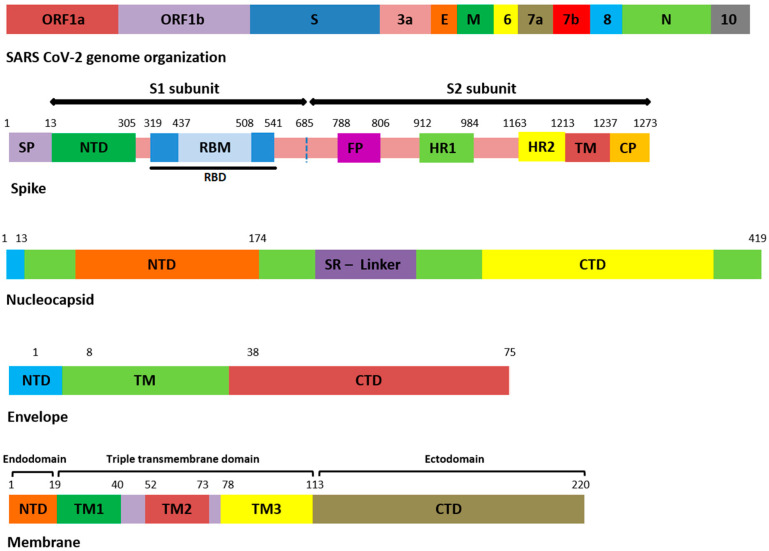
Domain organization of structural proteins. ORF: open reading frame, E: envelope, M: membrane, N: nucleocapsid, SP: signal peptide, NTD: N-terminal domain, RBD: receptor-binding domain, RBM: receptor-binding motif, FP: fusion peptide, HR: heptapeptide repeat sequence, TM: transmembrane, CP: cytoplasmic domain, SR: serine rich, CTD: C-terminal domain.

All the genomic sequences harbored 207 different non-synonymous mutations, including indel, among which 19 were unique to GISAID (S1). All the genomic samples were of GK clade. Mutations E554K (S1 domain of S), K1086Q, and C1250W (S2 domain of S) (Figure 1) were unique to S protein, and in GISAID, present in samples 1, 11, and 19. The remaining unique mutations in all samples are provided in the Supplementary File S1. 

### 3.4. SARS-CoV-2 Variants

All the samples were detected as Delta variants. Mutation details in S and other structures are provided in Supplementary Files S1–S3. These sequences harbored diverse kinds of mutations in the N-terminal and RBD of S protein that may upsurge the immune evasion possibility of Delta variant.

### 3.5. Mutations in Spike and Other Structural Proteins

Among the 207 mutations, 26 were detected in S protein (Supplementary File S2). The most common non-synonymous and indel mutations, present in all complete genome samples were spike_T478K, spike_T19R, spike_L452R, spike_F157del, spike_E156G, spike_P681R, spike_D614G, spike_R158del, and spike_G142D (Table 3). Some of these mutations were present in receptor-binding domain (RBD) of S protein (Figure 1). Mutations, spike_D950N and spike_T95I were present in twelve samples each.

**Figure 2:**
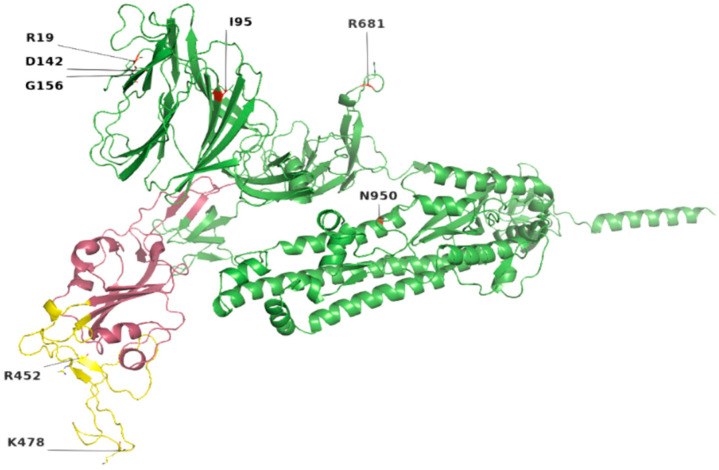
Location of most common mutations in S protein. Yellow indicates the receptor-binding motif, present in the RBD region (raspberry color). The majority of these mutations were found in the loop regions.

Similarly, nucleocapsid (N) also harbored nine different kinds of mutations (Supplementary File S2). Among these, the most common were N_D377Y, N_R203M, and N_D63G have been detected in all 18 samples (Table 3). The D63G was present in N-terminal domain (NTD) of N protein, which is also called RNA-binding domain (RBD) (Figure 1 and Figure 2). Two mutations, N_R203M and N_G215C, were present in SR-Linker region of N proteins and one D377Y in C-terminal domain (CTD). No mutation was detected in the envelope the protein where the membrane (M) protein harbored only two non-synonymous mutations (I82T (N = 18) and V70F (N = 1)) in the transmembrane domain. 

**Figure 3:**
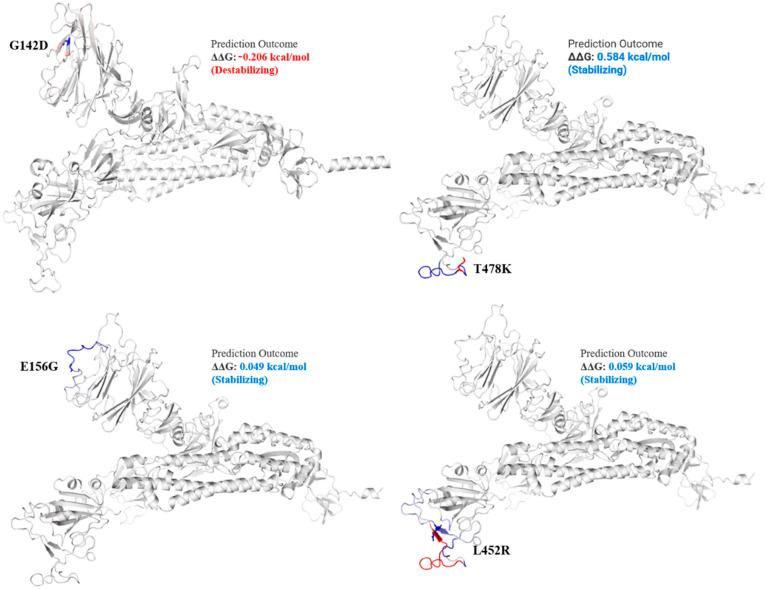
Mutation effect on S protein structure stability and flexibility. Mutants G142D, T478K, E156G, and L452R in S protein, exhibited stabilizing effect. The blue region shows rigidification of structure behind mutations and red shows gain in flexibility.

**Figure 4:**
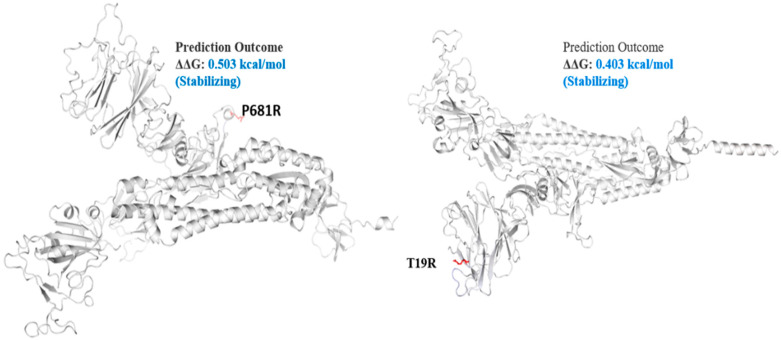
Mutation effect on S protein stability and flexibility. Mutants P681R and T19R in S protein, exhibited a stabilizing effect. The blue region shows rigidification of structure behind mutations and red shows gain in flexibility.

**Figure 5:**
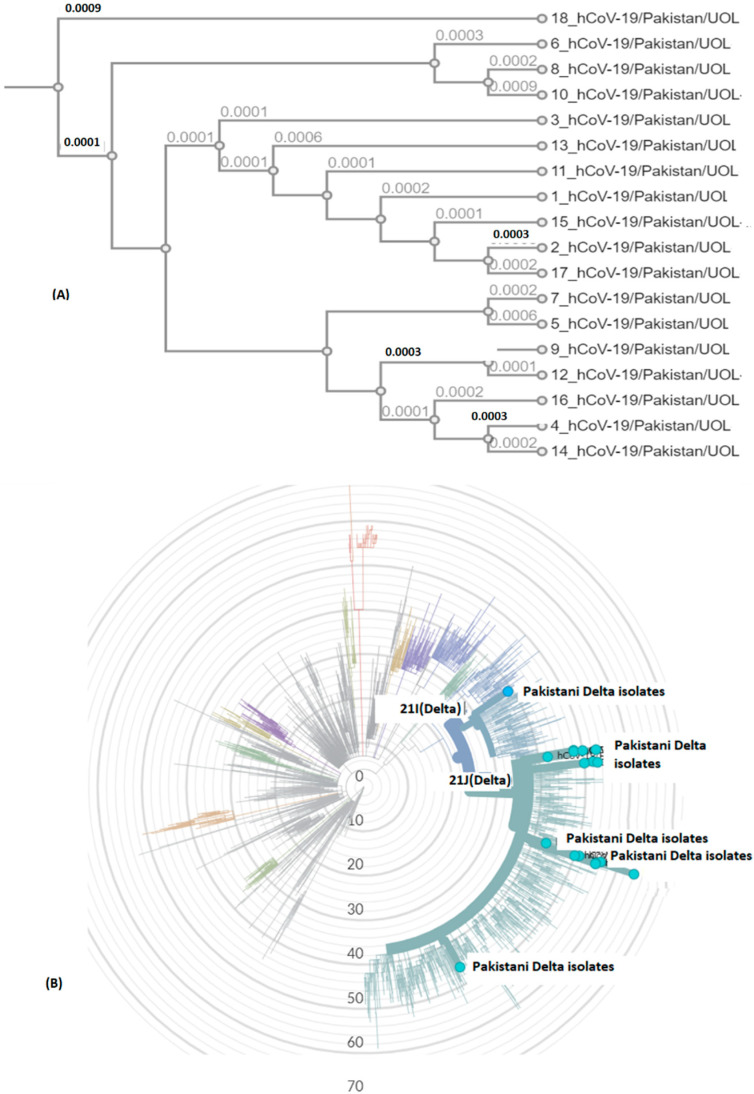
SARS-CoV-2 genomic epidemiology from Pakistan. The graph shows an estimate of divergence over the time period within genomes. (**A**) Current study isolates (labelled Pakistan Delta isolates). (**B**) Position of current SARS-CoV-2 isolates in radial tree (UOL) was built using Nextstrain 28 (Nextclade (nextstrain.org). Sub-clades have been color coded in the tree, characterized by some specific mutations in structural proteins (https://covariants.org/variants) (accessed on 28 September 2021).

Delta variant harbors L452R, P681R, and T478K in S protein. These mutations, along with others, present in all genomic samples, exhibited a stabilizing effect on the S protein structure (Figure 3 and Figure 4), and may demonstrate a good binding affinity towards human ACE2 SARS-CoV-2 receptor protein. Phylogenetically, all the isolates were 21I and 21J, subclades of Delta variants (Figure 5).

The spike_G142D mutant exhibited a little destabilizing affect (0.206 kcal/mole when compared with T478K (0.584 kcal/mol), E156G (0.049 kcal/mol), L452R (0.059 kcal/mol), P681R (0.503 kcal/mol), and T19R (0.403 kcal/mol). 

### 3.6. Unique Mutations in Structural Proteins

The sequences harbored 129 mutations in NSP in which 13 were unique to GISAID (Table 4) (Supplementary File S3). The highest number of unique mutations were detected in NSP3 (N = 10), followed by NSP5 (V86L), NSP12 (F313Y), and NS3 (I118L). The remaining unique mutations in the samples are provided in Supplementary File S3. 

### 3.7. Mutations in NSP

The mutation details in NSP are shown in Table 5. In SARS-CoV-2, NSP3 is a large multidomain protein of 1945 amino acids containing a papain-like protease (PLpro) domain (aa746-1060). PLpro, an essential component of the virus replication transcription complex, is highly conserved, present between unique and a nucleic acid-binding domains. Among the unique mutation, NS3_I118L was detected in 14 isolates (Table 4).

**Figure 6:**
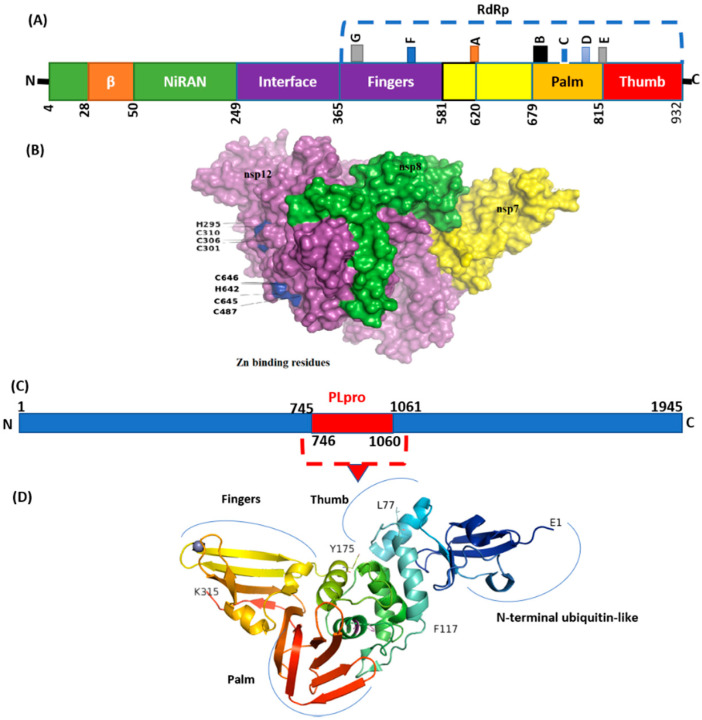
Structure of NSP12 and NSp3 (PLpro). (**A**) SARS-CoV-2 RdRp (PDB ID: 6M71) contains an N-terminal β-hairpin (residues 31–50). NiRAN (residues 50–249), interface domain (residues 251–365). The NiRAN domain is comprised of three helices and five β-strands, associated with RdRp domain (residues 366–920). (**B**) Complex of NSP12, NSP7, and NSP8. (**C**) Organization of NSP3 protein. (**D**) Structure of PLpro with mutations labeled NSP3_P822L (P77L), NSP3_L862F (L117F), and H920Y (H175y). PLpro has a small N-terminal ubiquitin-like (Ubl) domain and catalytic domain (thumb–palm–fingers).

The highest frequency of mutations was detected in virus NSP3 (Table 2) in which P1469S (N = 17), A488S (N = 17), and P1228L (N = 17) were the most common. The majority of these mutations were detected in the C-terminal domain (CTD) of NSP3. Deletions with a single frequency in amino acid regions 1500 to 1533 were also observed at CTD. However, the PLpro domain (746–1060) seems highly conserved (Figure 6). Mutations with a single frequency at position L862F, P822L, and H920Y were detected in the PLpro domain of NSP3 (Figure 6D). NSP2, which modulates the host cell survival signaling pathway, also harbored six non-synonymous mutations with a very low frequency. NSP4 harbors four mutations in which V167L in NTD and T492I in CTD were present in 17 isolates. 

NSP5 (main protease), harbored three non-synonymous mutations in a very low frequency. Four mutations were detected in NSP6 in which T77A was detected with the highest frequency (N = 17). Virus NSP12, which is also called RdRp (RNA-dependent RNA polymerase), plays an essential role in replication. Mutations NSP12_G671S (N = 18) and NSP12_P323L (N = 16) were the most common, present in the interface and RdRp domain (Figure 6). 

A single mutation P77L in NSP13 was detected in all genomic isolates (N = 18). Forty-one mutations were detected in NSP14, including five non-synonymous mutations in which NSP14_A394V (N = 17) NSP14_M72I (N = 6) were the most common. 

## 4. Discussion

The recent emergence of different SARS-CoV-2 variants posed severe health issues. The α is one of the identified variants that first emerged in the UK and became one of the predominant lineages worldwide. Pakistan reported its first case with α variant in December 2020 [29], triggered S protein, and rapidly spread, leading to the third wave in Pakistan [30]. Molecular epidemiology is essential for better management of viral diagnosis and treatment. However, limited genomic data are available on other genetic lineages from all provinces of Pakistan. Further, frequency of mutations in all target’s proteins, including NSP, has not been investigated properly. Surveillance of the population mainly depends on characterizing the differences between reported, emerging, and non-VOC lineages in order to design efficient diagnostic methods, proper therapeutics, and the designing of effective vaccines. 

Among the different variants, the Delta variant of SARS-CoV-2 is more deadly and highly transmissible with severe disease signs and symptoms. This variant has been detected in 77 countries as of 24 June 2021 [31,32]. The UK has faced drastic effects from public health measures due to the Delta variant. To prevent the rapid transmission of the virus in the population, there is an urgent need to design more integrated molecular diagnostic systems with the implication for strict rules of quarantines for international travelers. 

In the current study, all the genomic samples were detected as Delta variants when analyzed on GISAID CoV-Surver on 15 to 20 September 2021, collected during the fourth wave of infection. Mutations T478K, T19R, L452R, F157del, E156G, P681R, D614G, R158del, and G142D were the most common, present in all genomics isolates. The T478K mutation present in RBD of S protein is involved in interaction with human ACE2 [33,34]. The T478K is unique to the SARS-CoV2 Delta variant, present in the epitope region of potent neutralizing monoclonal antibodies [35]. This point mutation exhibited a structural stabilizing impact on S protein (Figure 3). 

A previous study during the third wave from Pakistan reported that the Delta variant is prevalent in 45% of cases followed by β (46%) [36]. Very little information was provided on mutations in structural proteins while NSP mutations were not provided in detail. In the current study, comprehensive details of mutations in the virus, all targeting proteins, including accessory proteins, have been provided for better understanding of the variations in circulating isolates. 

Among the structural proteins, the S protein harbored two mutations in the RBD region (L452R and T478K), four in the NTD (T19R, G142D, Δ156–157 and R158del), and one at the furin-cleavage site (P681R) and S2 region (D950N), detected in 12 virus genome sequences (Table 3). This cluster was observed in Indian isolates in October 2020 [37], exhibiting higher pathogenicity than other variants [38]. The T478K mutation at the RBD region of S protein, fell near the E484K that facilitates antibody escape [39]. The L452R is an antibody-escaping mutation and the virus with this mutant is resistant to convalescent plasma and monoclonal antibody therapy [40]. The furin-cleavage site plays an important role in viral pathogenesis [41] and mutation analysis revealed that L452R and P681R increase the ACE2 binding and transmissibility. Consistent with previous study [42] in which variants harbor different mutations, it exhibited increased binding between S protein RBD region and ACE2 receptor. Similar to this previous study, mutants T19R, E156G, L452R, T478K, and P681R in S protein (Figure 3 and Figure 4) exhibited a stabilizing effect on protein structure, facilitating the binding affinity for more stable interactions with human ACE2. This stability effect may increase the virus transmission in populations.

Mutations Arg158 and Phe-157/del were also detected in all genomic isolates (Table 3), present in NTD of S protein. In a more recent study, E156G, Arg158, and Phe-157/del were found, causing rigidity and reduced flexibility, thus providing fitness advantage and immune escape [43]. 

The Delta S protein P681R mutation has an important role in the variant replacement of the α-to-Delta. The Delta S P681R mutation present at the furin-cleavage site exhibited stabilizing effects (Figure 4), separating the S1 and S2 regions of S protein. Mechanistically, the P681R mutation of Delta improved the full-length cleavage of S1 and S2, facilitated the virus cell surface entry and increased infection 16. These mutations must be regularly monitored in ongoing pandemics for surveillance and virus severity [44]. 

Considering the circulation of the Delta virus with specific mutations and a high transmissible rate, the Pakistani national health authority needs to take timely measures against it. Further, molecular epidemiological studies with insight into genomic analysis of the virus are needed to screen the most prevalent variants and specific mutations that may affect the diagnosis and vaccine potency, to adopt potential measures in the future.

Emerging mutations in NSP may affect the virus transmission and pathogenicity. Recently, a synonymous mutation (F106F) in NSP3 along with other signature mutations exhibited a virus fitness effect [45]. In CoV-2 NSP3, the PLpro domain is a large protein and an essential member of replication transcription complex [46,47]. PLpro domain has a catalytic domain (Figure 1) for cleavage activity. Mutations in this domain may affect a catalytic process of PLpro. In the current study, three mutations were detected in this domain with many unique to GISAID. The effect of these mutations may be investigated in future studies for better designing of inhibitors against PLpro. 

The NSP6_L37F mutation, which was very common in the earlier infections, were associated with asymptomatic cases. NSP6 reduces autophagic ability, important for viral infections and promotes cell death [48]. Mutation NSP6_T77A in the current study (Table 2) seems emerging in the current wave. However, its effect on virus severity is needed to be explored for better management of COVID-19.

Previously, mutation P323L in RdRp was the most common in Pakistani isolates [49]. This mutation is still present in the Delta variants (Table 2), which has a stabilizing effect, while interacting with viral RNA, which lies in the interface domain of RdRp. This domain is the antiviral (filibuvir, Simeprevir) binding site [43], which may show weak interaction if mutations emerge. Mutation G671S in NSP12 detected in the current study has been observed as emerging, present in all 18 isolates, increasing the stability of the protein [50]. However, further investigations are needed to see its effect on virus pathogenicity. A single mutation, NSP13_P77L, which is characteristic only in Delta variants, was detected in all genomic sequences in the current study, and may have a destabilizing effect [51]. NSP14_A394V is the most common mutation in NSP14 (Table 2), and has a role in one of the positive section sites of virus [52]. 

Vaccine efficacy may be reduced in previous VOCs; however, BNT162b2 retained its potency against β VOC [53]. Omicron harbored a number of known and unique mutation patterns (Supplementary Files) as compared to other VoCs; therefore, the vaccines which are effective against Omicron infections are not clear. Vaccines have been found to be effective against other VOCs. Observational investigation in Qatar and Kaiser Permanente [54,55] found more than 90% vaccine efficacy against the Delta-variant. Data from New York indicates a good efficacy for individuals 65 years and older, exhibiting different levels of efficacy for different vaccines [56]. 

In conclusion, Delta variants with some unique mutations, are circulating during the fourth wave of the pandemic, in major cities in the Punjab province of Pakistan. The structural proteins and NSP harbored numerous mutations, present in different functional domains. Mutations spike_K1086Q, spike_E554K, and spike_C1250W were unique to GISAID. The most common mutations in the S protein exhibited a stabilizing effect, facilitating the binding affinity for S protein RBD with human ACE2. Geographic specific vaccines and drugs may be designed for better management of COVID-19 in the future. The geo-climate distribution of the mutations may decipher higher uniqueness and disease severity in underdeveloped countries, including Pakistan. The effect of these mutations on virus pathogenicity may be experimentally verified to access the effect on virus severity and vaccine efficacy. Continuous molecular epidemiology is required to screen geographic-specific mutations for better understanding of the virus pathogenicity, diagnosis, and treatment of COVID-19.

## Figures and Tables

**Table 1:** SARS-CoV-2 patients’ information.

Sample ID	Gender *	Age (Years)	Location
1	F	20	Okara
2	M	33	Sialkot
3	F	65	Sialkot
4	M	26	Sahiwal
5	M	31	Nankana
6	M	26	Shakot
7	M	60	Pak Pattan
8	F	25	Okara
9	M	24	Okara
10	M	28	Pak Pattan
11	F	43	Okara
12	M	46	Pak Pattan
13	F	42	Pak Pattan
14	M	53	Pak Pattan
15	M	30	Okara
16	M	65	Okara
17	F	21	Lahore
18	F	42	Lahore
19	M	42	Lahore
20	M	32	Okara

* F: female, M: male.

**Table 2:** Genome length and mutation’s pattern in SARS-CoV-2 sample.

Sample	Length #(nt)	Length #(aa)	Muts #	Muts %	Unique Muts
1	29,822	9705	37	0.38%	2
2	29,811	9705	34	0.35%	0
3	29,817	9705	38	0.39%	0
4	29,823	9705	31	0.32%	0
5	29,822	9705	37	0.38%	0
6	29,797	9705	33	0.34%	0
8	29,774	9705	36	0.37%	1
9	29,827	9705	32	0.33%	0
11	29,824	9705	31	0.32%	1
12	29,828	9705	35	0.36%	0
13	29,702	9669	69	0.71%	0
14	29,828	9705	28	0.29%	1
15	29,826	9705	34	0.35%	0
16	29,826	9705	31	0.32%	0
17	29,737	9705	37	0.38%	2
18	29,821	9705	39	0.40%	0
19	29,827	9705	36	0.37%	2
20	29,383	9650	98	1.02%	10

# Mut: mutations, nt: nucleotide, aa: amino acid.

**Table 3:** Mutational frequency in structural proteins.

Mutation	No. of Samples/Frequency
Spike_T478K	18
Spike_T19R	18
Spike_L452R	18
Spike_F157del	18
Spike_E156G	18
Spike_P681R	18
Spike_D614G	18
Spike_R158del	18
Spike_G142D	18
N_D377Y	18
N_D377Y	18
N_R203M	18
M_I82T	18
N_G215C	17
Spike_D950N	12
Spike_T95I	12

**Table 4:** Unique mutations in NSP and NS3 SARS-CoV-2 isolates from Punjab province, Pakistan.

Sample ID	Unique Mutations
20	NSP3_V1388A
20	NSP3_W1498S
20	NSP3_S1495N
20	NSP3_Y1535V
20	NSP3_S1534R
20	NSP3_K1497H
20	NSP3_S1494G
20	NSP3_D1499P
20	NSP12_F313Y *
19	NSP5_V86L *
14	NS3_I118L
8	NSP3_D339Y *
1	NSP3_T1303P

* NSP3; Papain-like protease (PLpro), NSP5; Main protease (Mpro), NSP12; RNA-dependent RNA polymerase.

**Table 5:** Mutations in NSP of SARS-CoV-2 circulating isolates in Punjab province.

Mutation	Count	Mutation	Count	Mutation	Count	Mutation	Count
NSP12_G671S #	18	NSP3_E391D	1	NSP3_F1516del	1	NSP14_I231del	1
NSP13_P77L	18	NSP3_M1529del	1	NSP3_I1514del	1	NSP14_V236del	1
NSP3_P1469S	17	NSP3_S1494G	1	NSP3_L1511del	1	NSP14_V136I	1
NSP3_A488S	17	NSP3_I1528del	1	NSP3_L1505del	1	NSP14_W247del	1
NSP3_P1228L	17	NSP3_F1503del	1	*NSP3_H920Y	1	NSP14_P203L	1
NSP4_T492I	17	NSP3_V1522del	1	NSP3_L1525del	1	NSP14_G248S	1
NSP4_V167L	17	NSP3_F1510del	1	NSP3_W1498S	1	NSP14_S221del	1
NSP6_T77A	17	NSP3_F1519del	1	NSP3_L1531del	1	NSP14_Q246del	1
NSP14_A394V	17	NSP3_A1512del	1	NSP3_L1500del	1	NSP14_S230del	1
NSP12_P323L	16	NSP3_A1526del	1	NSP4_A446V	1	NSP14_I242del	1
NSP14_M72I	6	NSP3_G1524del	1	NSP4_I377M	1	NSP14_Y235del	1
NSP3_S1370F	3	NSP3_K1497H	1	NSP5_S254F	1	NSP14_C216del	1
NSP3_S1285F	2	NSP3_D339Y	1	NSP5_V86L	1	NSP14_T223del	1
NSP5_P184T	2	NSP3_A1507del	1	NSP6_M92V	1	NSP14_D243del	1
NSP1_H83Y	1	NSP3_Y1521del	1	NSP6_T181I	1	NSP14_T219del	1
NSP2_L271F	1	NSP3_Y1535V	1	NSP6_V149A	1	NSP14_M241del	1
NSP2_E373K	1	NSP3_A1527del	1	NSP10_A20S	1	NSP14_P239del	1
NSP2_D315N	1	NSP3_Q1530del	1	NSP12_A16V	1	NSP14_W227del	1
NSP2_S591N	1	NSP3_S1534R	1	NSP12_A185V	1	NSP14_A225del	1
NSP2_A306V	1	NSP3_F1533del	1	#NSP12_L829F	1	NSP14_F240del	1
NSP2_K81N	1	NSP3_T1517del	1	NSP12_S318T	1	NSP14_Q245del	1
NSP3_S1495N	1	NSP3_R1518del	1	NSP12_F317Y	1	NSP14_C226del	1
NSP3_L1523del	1	NSP3_Y1513del	1	#NSP12_M380I	1	NSP14_H228del	1
NSP3_S1699F	1	NSP3_W1509del	1	NSP12_Q357H	1	NSP14_T215del	1
NSP3_E1508del	1	NSP3_D1499P	1	NSP12_F313Y	1	NSP14_G232del	1
NSP3_L862F *	1	NSP3_S211G	1	NSP14_D222del	1	NSP14_A220del	1
NSP3_P822L *	1	NSP3_V1506del	1	NSP14_F233del	1	NSP14_R213del	1
NSP3_A465V	1	NSP3_F1520del	1	NSP14_V244del	1	NSP14_N238del	1
NSP3_T1501del	1	NSP3_V1388A	1	NSP14_F217del	1	NSP14_S218del	1
NSP3_F1532del	1	NSP3_A644S	1	NSP14_D234del	1	NSP14_Y224del	1
NSP3_G1504del	1	NSP3_L1515del	1	NSP14_R212del	1	NSP14_Y237del	1
NSP3_A1502del	1	NSP3_T1303P	1	NSP14_H229del	1	NSP14_A214del	1

del: deletion, RdRp: RNA-dependent RNA-polymerase, NSP: non-structural protein. * Mutations in PLpro domain. # Mutations in RdRp domain of NSP12.

## Data Availability

Genomic data are freely available at NCBI GenBank BioProject PRJNA770504 (https://www.ncbi.nlm.nih.gov/bioprject/PRJNA770504) (Accession ID SRR16294858-SRR16294877).

## Supplementary Materials

The following supporting information can be downloaded at: https://www.mdpi.com/article/10.3390/genes13030552/s1, File S1: Frequency of mutations; File S2: Genome samples summary; File S3: Mutation details in all targets and samples.
